# Abnormal Discrepancy-Guided Knowledge Distillation for Image Anomaly Detection

**DOI:** 10.3390/jimaging12070291

**Published:** 2026-06-30

**Authors:** Zhenjun Yu, Lin Sun, Kai Wang, Fengxiang Jin

**Affiliations:** 1 College of Geodesy and Geomatics, Shandong University of Science and Technology, Qingdao 266000, China; yuzhenjun7@163.com (Z.Y.);; 2 Qingdao Star-Rising Technologies Co., Ltd., Qingdao 266000, China

**Keywords:** knowledge distillation, image anomaly detection, anomaly segmentation

## Abstract

Knowledge distillation is a cornerstone of image anomaly detection for amplifying subtle defects via teacher–student discrepancy, yet existing methods rely on feature alignment loss that causes reconstruction error confusion and degrades accuracy. To address this critical limitation, this study proposes an abnormal discrepancy-guided knowledge distillation method (DiffKD) that differentially guides student feature reconstruction through channel-level discrepancy masks, leveraging normal features as supervisory signals and abnormal discrepancy features as constraints to enhance anomaly detection performance. The approach integrates a knowledge distillation network for feature reconstruction with a segmentation network for anomaly localization, while utilizing prior anomaly samples and synthetic anomaly samples to provide real-time training data of anomalous samples. Extensive evaluations on the SUT-Crack and MVTec AD benchmarks validate the effectiveness and generalizability of our approach. On MVTec AD, it achieves 80.7% average precision (AP) and 81.9% instance-level average precision (IAP), showing competitive performance against the representative methods evaluated under the same protocol. These results not only demonstrate significant improvements in IAD accuracy but also highlight its promise for enabling real-time, automated anomaly detection in practical applications.

## 1. Introduction

Image anomaly detection aims to identify regions that deviate from normal visual patterns and is widely used in surface inspection, medical image analysis, and video surveillance [1,2]. In automated production, accurate real-time detection of subtle surface defects is essential for quality control. However, anomalous samples are scarce, diverse, and unpredictable, making fully supervised training difficult. This has motivated unsupervised methods that learn mainly from normal samples. Among existing anomaly detection methods, knowledge distillation has attracted attention because of its clear teacher–student mechanism [3,4]. Typically, a teacher network provides feature references, while a student network is trained on normal data. At inference, anomalous regions are expected to produce larger teacher–student discrepancies, which are then used for localization. Despite this progress, distillation-based methods still face two key limitations. First, a strong student network may also reconstruct some anomalous patterns, weakening the discrepancy signal and causing reconstruction-error ambiguity. Second, most methods do not explicitly use anomalous features during training, limiting their sensitivity to subtle but discriminative defects. In practice, the few available anomalous samples are therefore valuable but often underused.

To address these limitations, we propose DiffKD, an abnormal discrepancy-guided knowledge distillation framework. DiffKD constrains student reconstruction according to where and how anomalies deviate from normal features. A channel-level discrepancy mask identifies anomaly-responsive channels, while normal teacher features remain the reconstruction target. Therefore, the proposed method is described as few-shot anomaly-guided distillation: prior anomaly masks are used for training-stage sample generation and segmentation supervision, whereas no anomaly mask or prior sample is required during inference. The framework combines a distillation branch for feature learning, a segmentation branch for localization, and a sample generation module that uses both prior and synthetic anomalies. Experiments on SUT-Crack [5] and MVTec AD [6] evaluate its effectiveness, and ablation studies analyze the contribution of each component. The main contributions of this paper can be summarized as follows:

- We propose an abnormal discrepancy-guided knowledge distillation method that uses anomaly discrepancy features as guidance signals, improving sensitivity to subtle defects.
- We introduce a Channel-level Discrepancy Guidance Module (CDGM) to identify anomaly-responsive channels and impose differentiated constraints during feature reconstruction.
- We design a real-time sample generation strategy that combines prior anomaly objects with synthetic anomalies, improving training diversity under few-shot anomaly availability.
- Comprehensive evaluations on both SUT-Crack and MVTec AD benchmarks demonstrate the effectiveness of our method, achieving competitive performance compared with representative state-of-the-art techniques under controlled experimental settings.

The remainder of this paper is organized as follows. Section 2 reviews related work. Section 3 describes the proposed method and experimental settings. Section 4 reports the experimental results and ablation studies. Section 5 discusses limitations and efficiency, and Section 6 concludes the paper.

## 2. Related Work

Our framework is built upon the unsupervised anomaly detection paradigm, where normal data are used to train a teacher–student network, and a small number of prior anomaly samples are incorporated for guidance. To overcome overfitting caused by the scarcity of anomaly samples, we further adopt data augmentation strategies. Accordingly, this section presents a joint review of both unsupervised detection and data augmentation: the former provides the core discriminative logic for our method, while the latter offers feasible solutions to improve sample utilization efficiency.

### 2.1. Unsupervised Anomaly Detection

Unsupervised image anomaly detection involves developing models that learn the normative distribution of data from a training set containing only normal instances, without labeled anomalies, so that during testing they can effectively detect and localize samples or areas that represent a significant deviation from this norm. Existing unsupervised methods mainly include Normalizing Flow [7,8], Memory Bank [9], Multivariate Gaussian Distribution [10], and Knowledge Distillation [11,12]. Unsupervised methods based on knowledge distillation have garnered widespread attention in recent years, with numerous studies demonstrating their superior performance in industrial defect detection and medical image analysis by exploiting the representational gap between teacher–student architectures. Bergmann et al. [11]. introduced Uninformed Students, which employs multi-student ensemble and uncertainty modeling to detect anomalies via prediction discrepancies among student networks on teacher features. Subsequently, MKD [13] explicitly incorporates knowledge distillation, utilizing multi-resolution feature discrepancies for more precise anomaly detection and localization. Following MKD, researchers have continued to explore along multiple directions. On one hand, STPM [14] introduced a feature pyramid matching mechanism, enabling the student network to directly mimic the multi-scale feature outputs of the teacher network, significantly simplifying the training process and improving localization accuracy. Subsequently, RSTPM [15] addressed the overfitting issue of the student network to the teacher network in STPM by introducing a reconstruction branch, enhancing sensitivity to anomalous regions through image reconstruction constraints. DeSTSeg [16] further integrated denoising training with segmentation guidance, enhancing student robustness through synthetic noise and adaptively fusing multi-level teacher–student features via a segmentation network, leading to substantial performance improvements. Building upon DeSTSeg, KR-FP [17] introduced a knowledge review module to enhance cross-layer information interaction through attention fusion and hierarchical context loss, while replacing traditional image-level augmentation with feature-level perturbations to further improve robustness in complex scenarios. On the other hand, to address the non-discriminative feature issue caused by teacher–student network homogenization in MKD, RD4AD [12] proposed a reverse distillation architecture that compresses teacher features into a compact embedding, from which the student network reconstructs multi-scale features, significantly enhancing feature discrepancies in anomalous regions. DMDD [18] tackled the over-generalization problem of student networks by proposing a decoupled distillation framework that separates student features into normal and anomalous components, introducing dual-modeling distillation based on normal–abnormal image pairs, achieving excellent pixel-level AUC on MVTec AD. KD-KI [19] proposed a knowledge infusion mechanism that injects structured hierarchical knowledge from the teacher network into the student network, introducing feature deviation loss to prevent shallow networks from “shortcut” learning, achieving superior performance in small defect detection scenarios. CDA [20] introduced a cross-scale distillation paradigm, breaking through the traditional limitation of knowledge transfer only between same-scale feature pairs, while designing a global–local compression module to effectively reduce computational complexity. These methods have advanced the development of knowledge distillation in anomaly detection from various perspectives, collectively building an increasingly comprehensive technical system in this direction.

### 2.2. Data Augmentation

In the field of anomaly detection, data augmentation plays a pivotal role in establishing a clear decision boundary between normal and abnormal patterns by artificially generating diverse pseudo-anomalous samples, thereby effectively mitigating the scarcity of defective data and enhancing model generalization. In image anomaly detection, image-level data augmentation methods primarily generate diverse pseudo-anomalous samples through global image transformations or mixing operations. Early simple geometric transformations, such as random rotation, flipping, cropping, and color jittering, expand the training set by applying perturbations to normal samples, enhancing the model’s robustness to variations in illumination, angle, and other factors. Subsequently, mixing-based methods like Mixup [21] overlay the pixels of two images at a specific ratio and generate soft labels, enabling the model to learn smoother decision boundaries. Cutout [22] randomly erases rectangular regions and fills them with zero values to simulate occlusions or local missing parts, forcing the model to focus on broader contextual features. Cutmix [23,24] further combines the advantages of both approaches by replacing a rectangular region in one image with the corresponding region from another image, generating mixed samples with locally coherent semantics, thereby introducing diversity while preserving local structures. These image-level methods are computationally efficient and easy to implement, providing an effective preprocessing approach for alleviating the scarcity of anomalous samples. However, these image-level augmentation methods primarily rely on simple perturbations or mixing operations that may generate unrealistic or semantically inconsistent samples, failing to capture the complex structural characteristics of real-world anomalies and potentially introducing noise that confuses the decision boundary between normal and abnormal patterns. To generate more realistic anomalous samples, Generative Adversarial Networks (GANs) [25,26,27] and Variational Autoencoders (VAEs) [28,29] are widely used for data augmentation in anomaly detection, generating high-quality synthetic data by learning the distribution of normal or anomalous samples, thereby expanding the training set. However, GANs and VAEs often suffer from training instability, mode collapse, or limited reconstruction quality, which may lead to generated samples that lack diversity or fail to accurately represent the complex characteristics of real anomalies, thereby limiting their effectiveness in enhancing model robustness. Diffusion model-based anomaly generation has become a research hotspot in image anomaly detection, addressing the challenge of scarce real anomalies. AnomalyDiffusion [30] proposes an anomaly image generation framework based on few-shot learning. DualAnoDiff [31] further introduces a dual-correlated diffusion model, which generates complete images and anomalous regions through two mutually collaborative diffusion processes, enhancing the diversity and realism of generated anomalies. AnoGen [32] employs an anomaly-driven generation strategy, using only a small number of real anomaly samples to guide the diffusion model in generating diverse synthetic anomalies, thereby supporting the training of downstream detection models. DiAD [33] proposes a diffusion framework for multi-class anomaly detection, achieving precise localization of anomalous regions through the reconstruction process. However, challenges remain, including insufficient interpretability validation of generated samples and generation efficiency that falls short of meeting real-time industrial deployment requirements.

## 3. Methodology

**Figure 1:**
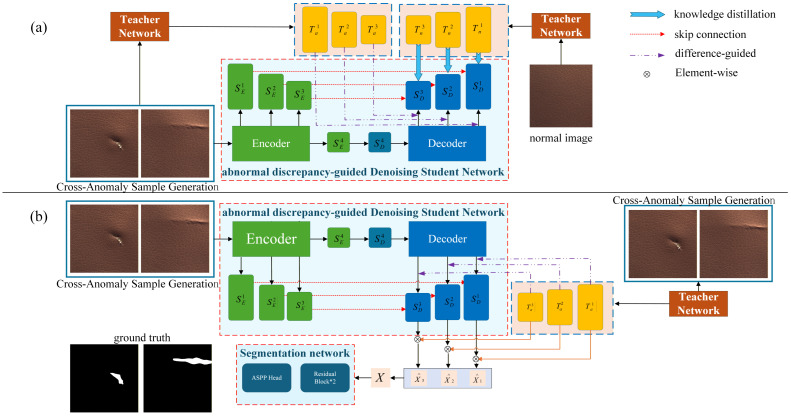
Overview of the proposed DiffKD framework. In the first step (**a**), the discrepancy-guided Denoising Student Network is trained on anomalous images to produce feature representations that closely match those of the teacher network when given normal images. In the second step (**b**), the element-wise product of the normalized outputs from both networks is concatenated and fed into the segmentation network, which is trained to generate the final anomaly score map.

Figure 1 shows the overall architecture of DiffKD. The framework contains three components: Cross-Anomaly Sample Generation (CASG), a knowledge distillation network, and an anomaly segmentation network. CASG generates training anomalies from prior anomaly objects and synthetic defects. The distillation network reconstructs normal teacher features, while channel-level discrepancy masks guide the student to preserve anomaly-sensitive responses. The segmentation network then predicts the anomaly score map from the resulting discrepancy features. Training has two stages: the discrepancy-guided denoising student is first optimized and frozen, and the segmentation network is then trained with CASG-generated masks. These masks are used only during training; at inference, the model receives only the test image and outputs an anomaly score map.

### 3.1. Cross-Anomaly Sample Generation (CASG)

**Figure 2:**
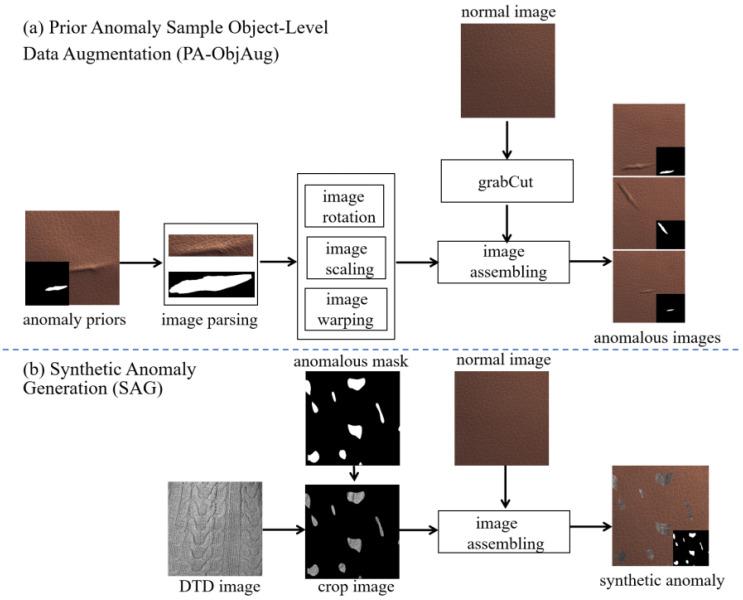
Cross-anomaly sample generation schematic diagram.

Cross-Anomaly Sample Generation (CASG) uses limited known anomalies while preserving diversity for unseen defect types. It contains two components: prior anomaly object-level augmentation (PA-ObjAug) and synthetic anomaly generation (SAG). During training, CASG samples from these two sources online according to a mixing ratio. The mechanism is shown in Figure 2, and the training set is defined in Equation (1).

(1) $$D_{\mathrm{train}}={\left\{(x_{i},y_{i})\right\}}_{i=1}^{N}={\left\{(x_{j}^{\mathrm{aug}},y_{j})\right\}}_{j=1}^{N}\cup {\left\{(x_{k}^{\mathrm{syn}},y_{k})\right\}}_{k=1}^{N}$$

where $x_{i}$ denotes the *i*-th training image and $y_{i}$ is its corresponding mask. ${\left\{(x_{j}^{\mathrm{aug}},y_{j})\right\}}_{j=1}^{\lambda N}$ represents a subset of λN augmented samples generated from prior anomaly instances. The superscript aug indicates that these samples are derived through PA-ObjAug techniques. We set λ=0.5 by default so that PA-ObjAug and SAG are sampled with equal probability. This balances realistic defect morphology from PA-ObjAug with distributional diversity from SAG. A sensitivity analysis of the λ parameter is conducted in Section 4.4.2.

#### 3.1.1. Prior Anomaly Sample Object-Level Data Augmentation (PA-ObjAug)

PA-ObjAug consists of image parsing, anomalous object augmentation, and image assembling. First, a prior anomaly image is parsed into anomaly objects and masks. The objects are then transformed and inserted into normal images to synthesize training images and masks.

During image parsing, a dot product operation is first applied between the prior anomalous sample and its corresponding ground-truth mask; the resulting masked region is then cropped to obtain each object $X_{p}^{k}$ and its corresponding ground truth $M_{p}^{k}$, as illustrated in Equation (2).

(2) $$\left\{X_{p}^{k},M_{p}^{k}|p<5,k>0\right\}=f_{c}\left(f_{d}\left(X_{p},M_{p}\right)\right)$$

where $X_{p}^{k}$ and $M_{p}^{k}$ denote the *k*-th anomaly object and its mask from the *p*-th prior anomaly sample. The default setting uses four representative prior anomaly samples per anomaly type. These prior samples are removed from the evaluation set to prevent any overlap between training guidance and testing. Given that this decision may influence performance, the effect of different numbers of prior anomaly samples is assessed in Section 4.4.1.

To increase anomaly diversity, each extracted object is transformed along three dimensions—angle, scale, and shape, as defined in Equation (3).

(3) $$X_{pA}^{k},M_{pA}^{k}=f_{\mathrm{aug}}\left(X_{p}^{k},M_{p}^{k},T,v_{\min},v_{\max}\right)$$

where $X_{pA}^{k},M_{pA}^{k}$ denote the augmented anomalous data and the corresponding mask, respectively. Parameter *T* represents any one of the three dimensions, and $v_{\min},v_{\max}$ denote the minimum and maximum variation ranges for the selected dimension. In practice, a dimension is first randomly chosen, and then the transformation parameters are randomly sampled within its corresponding range to simultaneously alter both the anomalous target and its mask.

In the image assembling stage, Grabcut [34] is first applied to rapidly extract foreground objects from the collected normal samples. Subsequently, the augmented anomalous data and the extracted foreground regions are randomly assembled, generating both the training sample and its corresponding mask required for model training.

#### 3.1.2. Synthetic Anomaly Generation (SAG)

Since real anomalies are diverse and incomplete, SAG supplements PA-ObjAug with synthetic defects. A Perlin-noise mask is generated and binarized, a texture patch is sampled from an external dataset such as DTD, and the patch is blended into a normal image with opacity sampled from [0.15, 1]. The process is defined in Equation (4).

(4) $$x_{k}^{\mathrm{syn}}=\beta \left(M⊙A\right)+\left(1-\beta \right)\left(M⊙I_{n}\right)+\left(1-M\right)⊙I_{n}$$

where $x_{k}^{\mathrm{syn}}$ denotes the *k*-th synthetic anomaly, *M* is the anomaly mask, *A* is a texture patch from an external dataset such as DTD, $I_{n}$ is the normal image, and β is the opacity coefficient.

During training, CASG uses λ to balance PA-ObjAug and SAG. In each iteration, a random value is sampled from [0, 1]. If it is smaller than λ, PA-ObjAug is used; otherwise, SAG is used.

**Figure 3:**
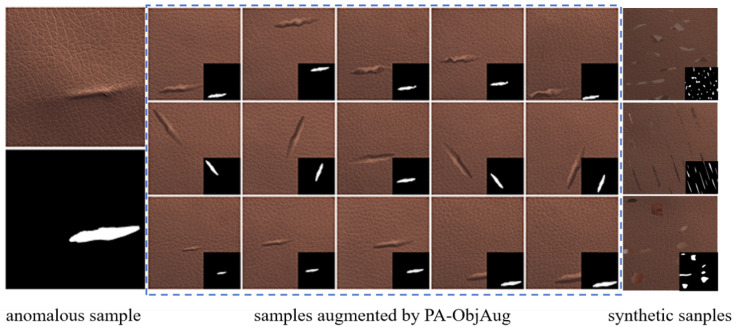
Examples of anomalous samples generated by CASG.

Figure 3 shows examples generated by CASG. PA-ObjAug produces variants from real prior anomalies by changing orientation, scale, and morphology, while SAG provides irregular synthetic defects from Perlin-noise masks and external textures. Together, they improve anomaly diversity in the CASG.

### 3.2. Knowledge Distillation Network

The knowledge distillation network contains a teacher network and an abnormal discrepancy-guided denoising student network, as shown in Figure 1. Its goal is to reconstruct normal teacher features while preventing anomalous regions from being reconstructed too well. The student adopts an encoder–decoder structure. Normal teacher features provide the reconstruction target, whereas anomaly-derived discrepancies provide guidance through a channel-wise mask. This asymmetric design reduces reconstruction-error ambiguity and improves anomaly discriminability.

#### 3.2.1. Teacher Network

The teacher network employs a ResNet-18 pre-trained on ImageNet, with the fourth residual block (Block4) removed to leverage the multi-scale semantic information extracted by the remaining architecture. Block4 is removed because the proposed segmentation task relies more strongly on mid-level structural and textural cues than on highly compressed category-level semantics; retaining the first three residual stages also reduces computational cost while preserving feature maps with sufficient spatial resolution. This modified backbone provides knowledge transfer targets for the discrepancy-guided denoising student network, ensuring that the distilled student network not only retains the feature extraction capability of the teacher but also maintains the integrity of the feature pyramid hierarchy. For an input image, the teacher network generates three feature maps at resolutions of 1/4, 1/8, and 1/16 of original input. To enable the abnormal discrepancy-guided denoising student network to reconstruct features consistent with the normal features extracted by the teacher while avoiding similarity to anomalous features, the teacher network is designed to fulfill dual roles: normal features are used as explicit distillation targets, whereas anomalous features are used only to construct discrepancy constraints and are not treated as reconstruction targets. Consequently, the teacher network extracts different types of features depending on the training stage. During the training of the discrepancy-guided denoising student network, the teacher network needs to extract multi-scale semantic information from normal samples for knowledge transfer to the student, while also extracting multi-scale semantic information from anomalous samples to guide the student’s feature reconstruction process. During the training of the segmentation network, the teacher network only needs to extract semantic information from anomalous samples to compute the discrepancy with the features reconstructed by the student network, thereby meeting the input feature requirements of the segmentation network. Throughout the entire training process, the feature maps extracted by the teacher network are denoted as:

(5) $$F_{T}=\left\{T_{n}^{i},T_{a}^{i}|i=1,2,3\right\}$$

where $T_{n}^{i}$ denotes the deep features of the normal sample at the i-th layer, and $T_{a}^{i}$ denotes the deep features of the anomalous sample at the i-th layer.

#### 3.2.2. Abnormal Discrepancy-Guided Denoising Student Network

**Figure 4:**
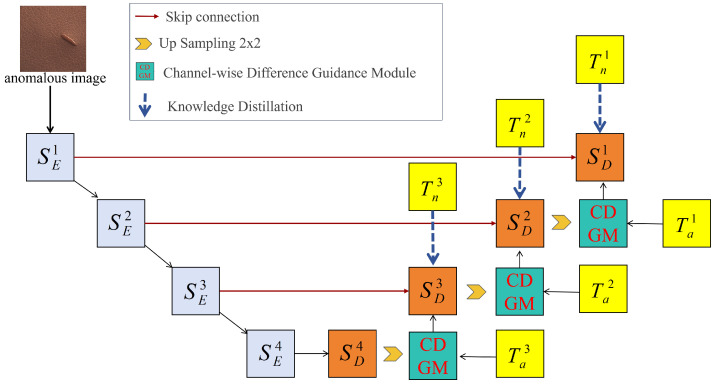
Architecture diagram of the abnormal discrepancy-guided denoising student network.

The abnormal discrepancy-guided denoising student network uses a symmetric encoder–decoder architecture, as shown in Figure 4. The encoder adopts a ResNet-18 backbone and extracts feature maps $S_{E}^{1}$, $S_{E}^{2}$, $S_{E}^{3}$, and $S_{E}^{4}$. In the decoder, the deepest feature $S_{E}^{4}$ is reconstructed directly, while CDGM is inserted into the reconstruction of $S_{D}^{1}$, $S_{D}^{2}$, and $S_{D}^{3}$. Normal teacher features $T_{n}^{1}$, $T_{n}^{2}$, and $T_{n}^{3}$ serve as distillation targets, whereas anomalous features $T_{a}^{1}$, $T_{a}^{2}$, and $T_{a}^{3}$ are used to compute discrepancy guidance. This design separates the supervision target from the guidance signal: the student decoder is optimized to match normal teacher features, while anomaly-derived discrepancies modulate the reconstruction pathway. Equation (6) defines the hierarchical decoder reconstruction.

(6) $$\left\{S_{D}^{i}\right\}=\left\{SK\left(S_{E}^{i},S_{fused}^{i+1}\right)|i=1,2,3\right\}=\left\{SK\left(S_{DU}^{i},T_{a}^{i}\right)|i=1,2,3\right\}$$

where SK indicates the residual connection module, and $S_{fused}$ refers to the channel-wise discrepancy guidance module for the i-th layer.

The corresponding multi-scale teacher–student layer mapping and CDGM placement are further summarized in Table 1.

CDGM uses anomalous teacher features to construct channel-wise guidance for student reconstruction. It first computes magnitude discrepancy $\Delta amp^{i}$ and cosine discrepancy $\Delta cos^{i}$ between the anomalous teacher feature and the upsampled student decoder feature, as shown in Equation (7). The discrepancy is used as a guidance mask rather than as a loss target. The fused mask $diff_{mask}^{i}$ is defined in Equation (8), weighted by channel attention in Equation (9), and applied to obtain discrepancy-guided features in Equation (10). The weighted mismatch mask notation is kept consistent between Equations (9) and (10).

(7) $$\begin{array}{c} \Delta amp^{i}=\left\{L2_{norm}\left(\left|T_{a}^{i}-S_{DU}^{i}\right|\right)|i=1,2,3\right\}\\ \Delta cos^{i}=\left\{1-\cos\left(T_{a}^{i},S_{DU}^{i+1}\right)|i=1,2,3\right\} \end{array}$$

(8) $$diff_{mask}^{i}=\alpha \cdot \Delta amp^{i}+\left(1-\alpha \right)\cdot \Delta cos^{i}$$

(9) $$W_{\mathrm{mask}}^{i}={\mathrm{diff}}_{\mathrm{mask}}^{i}\times \mathrm{CA}\left({\mathrm{diff}}_{\mathrm{mask}}^{i}\right)$$

(10) $$G^{i}=S_{\mathrm{DU}}^{i+1}\times \left(1+W_{\mathrm{mask}}^{i}\right)$$

where α is set to 0.5 and analyzed in the ablation study. $S_{DU}^{i+1}$ is the upsampled feature map from the next decoder layer, aligned with $S_{D}^{i}$. The channel attention module CA(.) recalibrates channel-wise feature responses.

CDGM further introduces a learnable parameter δ to balance the original feature $S_{DU}^{i+1}$ and the discrepancy-guided feature $G_{i}$, as formulated in Equation (11).

(11) $$S_{\mathrm{fused}}^{i}=\delta G^{i}+\left(1-\delta \right)S_{\mathrm{DU}}^{i+1}$$

The student decoder outputs $S_{D}^{1}$, $S_{D}^{2}$, and $S_{D}^{3}$ are channel-aligned with the corresponding teacher features $T_{n}^{1}$, $T_{n}^{2}$, and $T_{n}^{3}$. The multi-scale discrepancy features ${\hat{X}}_{1}$, ${\hat{X}}_{2}$, and ${\hat{X}}_{3}$ are then upsampled to a common spatial size and aggregated into $\hat{X}$ for the segmentation network.

During the training of the abnormal discrepancy-guided denoising student network, normal samples are processed by the fixed teacher network to extract their feature representations, while the cross-generated anomalous samples are fed into the student network for feature reconstruction. In the training process, the optimization loss is used only between the normal teacher features and the reconstructed student features. Anomalous teacher features participate in CDGM mask construction, but they do not serve as direct supervision targets in the distillation loss. As shown in Equations (12) and (13), the distillation loss sums the cosine distances between normal teacher features $T_{n}^{i}$ and reconstructed student features $S_{D}^{i}$ over three feature levels.

(12) $$L_{\cos}=\sum_{i=1}^{3}\left(\frac{1}{W_{i}H_{i}}\sum_{r=0}^{W_{i}}\sum_{c=0}^{H_{i}}D_{i}\left(r,c\right)\right)$$

(13) $$D_{i}\left(r,c\right)=1-\sum_{c}^{C_{i}}\frac{F_{T_{n}^{i}}\left(r,c\right)⊙F_{S_{D}^{i}}\left(r,c\right)}{{∥F_{T_{n}^{i}}\left(r,c\right)∥}_{2}{∥F_{S_{D}^{i}}\left(r,c\right)∥}_{2}}$$

Here, (r,c) denotes a spatial coordinate, and $F_{T_{n}^{i}}\left(r,c\right)$ and $F_{S_{D}^{i}}\left(r,c\right)$ denote the normal teacher activation and reconstructed student activation at layer *i*, respectively.

### 3.3. Segmentation Network

**Figure 5:**
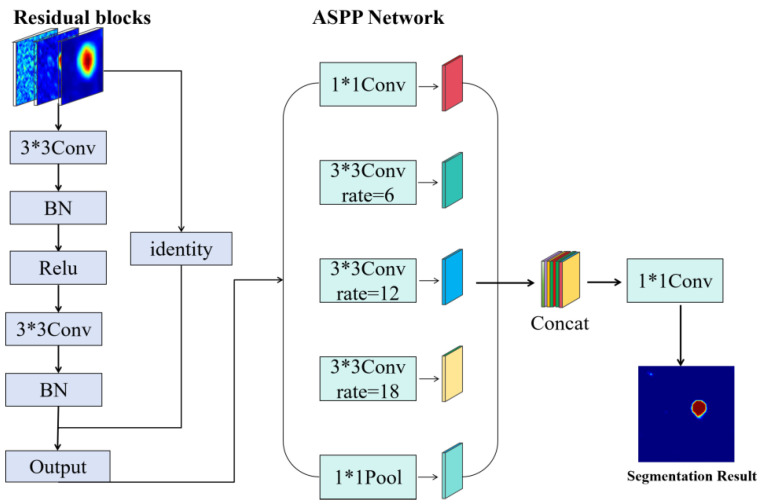
Architecture of the anomaly segmentation network. The symbol “*” denotes the convolution operation, and different colors are used only to distinguish different network modules.

The architecture of the anomaly segmentation network is illustrated in Figure 5. It comprises a residual network featuring two residual blocks, followed by an Atrous Spatial Pyramid Pooling (ASPP) module designed for multi-scale feature extraction. The ASPP module integrates five parallel branches: a 1×1 convolution for local feature extraction, three 3×3 dilated convolutions with rates of 6, 12, and 18 to accommodate anomalies of varying scales, and a global average pooling branch that supplies global contextual information, thereby compensating for the limited receptive field of the convolutional layers.

During the training of the segmentation network, both the teacher and student networks are frozen. The training samples, along with their corresponding ground-truth masks, serve as the input to the distillation framework and the supervision for segmentation, respectively. The pre-computed differential feature set $\hat{X}$ is fed into the segmentation network, which outputs a probability map $\hat{Y}$. As formulated in Equation (16), the overall loss function for the segmentation network combines a focal loss and L1 loss, which are designed to balance the trade-off between precision and recall and defined in Equations (14) and (15) respectively.

(14) $$L_{focal}=-\frac{1}{H_{1}W_{1}}\sum_{r,c=1}^{H_{1}W}{\left(1-p_{rc}\right)}^{\gamma }\log\left(p_{rc}\right)$$

(15) $$L_{l1}=\frac{1}{H_{1}W_{1}}\sum_{r,c=1}^{H_{1}W}\left|M_{rc}-{\hat{Y}}_{rc}\right|$$

(16) $$L_{seg}=L_{focal}+L_{l1}$$

where γ denotes the focusing parameter. For a feature map $\hat{Y}$ of spatial dimensions $H_{1}$ × $W_{1}$, we denote by $p_{rc}$ the probability that the pixel located at (r,c) is classified as foreground (anomaly). $p_{rc}$ is defined as Equation (17).

(17) $$p_{rc}=M_{rc}{\hat{Y}}_{rc}+\left(1-M_{rc}\right)\left(1-{\hat{Y}}_{rc}\right)$$

where $M_{rc}$ is the ground-truth mask.

During inference, the input images are simultaneously fed into both the teacher network and the abnormal discrepancy-guided denoising student network. The teacher network extracts authentic feature representations, while the student network reconstructs features corresponding to normal patterns. The feature discrepancies between their outputs are subsequently computed and passed to the segmentation network, which performs accurate anomaly localization and segmentation.

### 3.4. Dataset

To comprehensively evaluate the anomaly detection performance of the proposed method, we conduct experiments on two publicly available datasets: SUT-Crack and MVTec AD. Both qualitative and quantitative analyses are performed to compare the proposed approach against existing methods.

The SUT-Crack dataset is a pavement crack dataset comprising 130 annotated images originally designed for deep learning-based classification and segmentation tasks. To adapt this dataset to our model, each image is randomly cropped into 512×512 patches, which are then categorized as either cracked or non-cracked. To prevent image-level leakage, the original images are first divided into disjoint training and test groups, and patch extraction is then performed separately within each group. Therefore, patches cropped from the same original image never appear simultaneously in training and evaluation. The training set consists of 600 non-cracked (i.e., “normal”) patches, while the evaluation and test sets comprise 270 cracked patches.

The MVTec AD dataset serves as a benchmark for image anomaly detection, encompassing over 70 defect types, which exhibits significant pixel-level class imbalance, with normal regions accounting for 97.26% of pixels and anomalous regions occupying merely 2.74%. Moreover, anomaly scales vary dramatically, ranging from minute defects covering less than 0.1% of the image area to large-scale faults exceeding 10%. These characteristics, including blurred anomaly boundaries, high defect diversity, and extreme anomaly sparsity, define the core challenge of achieving precise localization and recognition.

Meanwhile, since four samples were selected as prior anomaly samples for each anomaly during the training process, to ensure the accuracy of the experiment, these samples were excluded from the accuracy evaluation, and all other samples in each dataset were included in the performance assessment.

### 3.5. Training Configuration

All experiments were conducted using Python 3.9 and the PyTorch 2.6.0. framework. Input images were uniformly resized to 256×256 pixels to ensure consistent experimental conditions across all trials. To constrain model capacity and mitigate overfitting, $\ell_{2}$ regularization with a weight decay coefficient of $1\times 10^{-4}$ was applied uniformly to all optimizers throughout the entire training pipeline.

For the abnormal discrepancy-guided denoising student network, training was performed with a batch size of 16 and an initial learning rate of 0.4 for 2000 iterations [16]. An automatic learning rate decay strategy was integrated to suppress overfitting and accelerate convergence. The Stochastic Gradient Descent (SGD) optimizer (learning rate =0.4, momentum =0.9, weight decay $=1\times 10^{-4}$) was used to optimize only the student network, while the segmentation network was frozen to reduce the optimization search space and eliminate interference.

When training the segmentation network, the batch size remained 16, while the number of iterations was increased to 6000 and the initial learning rate was adjusted to 0.1 to accommodate the higher complexity of the segmentation task and ensure sufficient feature learning. The SGD optimizer (momentum =0.9, weight decay $=1\times 10^{-4}$) was retained, and the denoising student network was frozen to prevent unintended parameter drift. A layer-wise differential learning rate strategy (Residual blocks: 0.1, ASPP head: 0.01) was adopted to enable precise parameter updates and balance feature extraction with segmentation accuracy.

## 4. Experimental Results

### 4.1. Evaluation Metrics

We use image-level, pixel-level, and instance-level metrics. Image-level AUC [35] evaluates anomaly classification across thresholds. Pixel-level AUC and average precision (AP) [36] measure pixel-wise separability and precision–recall quality. Per-region overlap (PRO) measures segmentation completeness, while instance average precision (IAP) treats each connected anomaly as an instance and requires at least 50% pixel recall for a valid detection. We also report IAP@90 to evaluate precision under a high-recall condition.

### 4.2. Performance Comparison with State-of-the-Art Models

We evaluate DiffKD through qualitative visualization, quantitative comparison with representative baselines, and ablation studies. In all heatmaps, warmer colors indicate stronger anomaly responses, whereas cooler colors indicate weaker responses or normal regions.

#### 4.2.1. Qualitative Analysis of Detection Results

**Figure 6:**
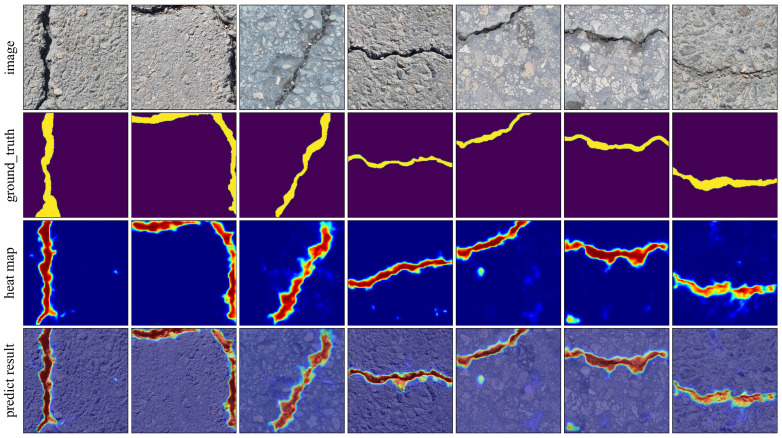
Anomaly detection results on SUT-Crack.

**Figure 7:**
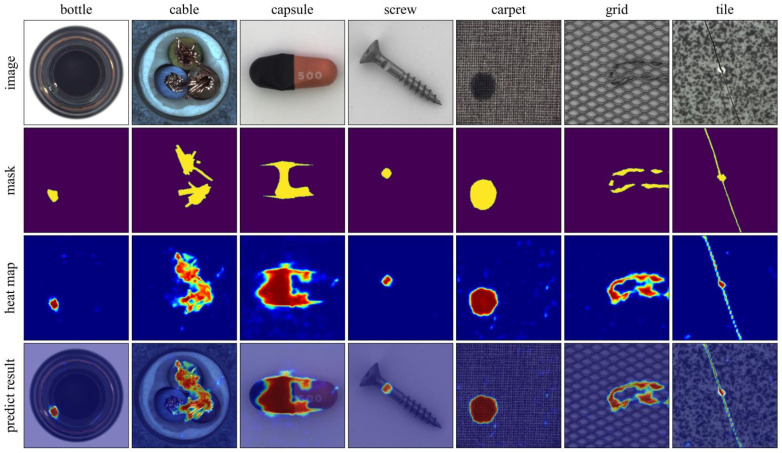
Anomaly detection results on MVTec AD.

Figure 6 and Figure 7 respectively present the detection performance of the proposed method on the SUT-Crack and MVTec AD datasets. For clear and intuitive visualization, all results are organized in a consistent layout: original image, ground-truth anomaly mask, predicted anomaly heatmap, and final localization result, enabling straightforward comparison between model outputs and manual annotations.

Figure 6 shows results on SUT-Crack, which contains thin, irregular cracks under complex backgrounds. The heatmaps produced by DiffKD localize most crack regions and preserve their linear structure, indicating good sensitivity to small defects.

Figure 7 shows representative MVTec AD results. The first four columns are object categories, and the last three are texture categories. For object defects such as deformation, breakage, and contamination, DiffKD produces concentrated responses around defective regions. For texture defects such as carpet stains, grid fractures, and tile cracks, the heatmaps suppress most background responses and highlight local irregularities.

Overall, the visual results suggest that DiffKD can handle both structural and textural anomalies, although quantitative results are still needed for a complete comparison.

#### 4.2.2. Comparative Experiments with Mainstream Methods

**Figure 8:**
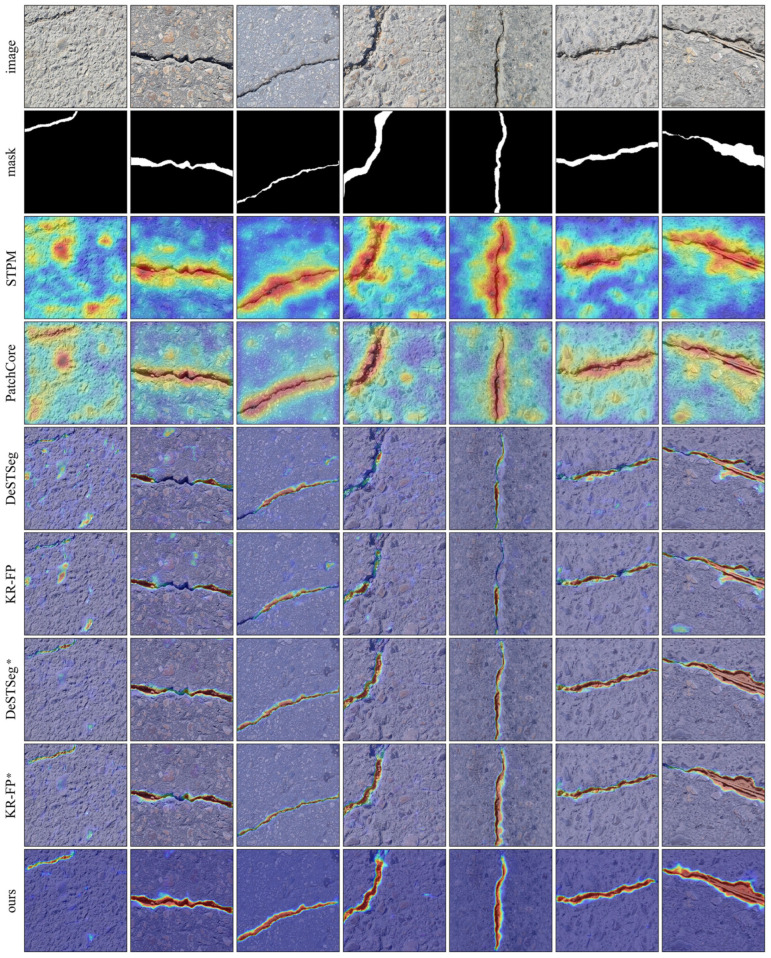
Comparison of anomaly detection performance on the SUT-Crack dataset.

**Figure 9:**
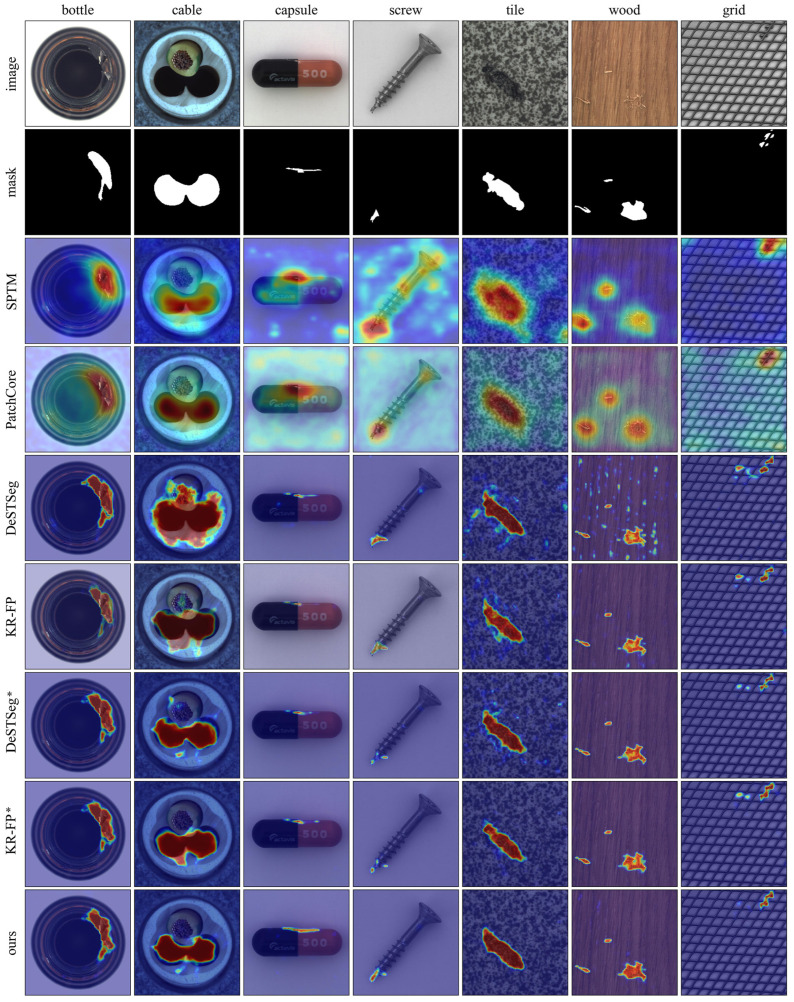
Comparison of anomaly detection performance on the MVTec AD dataset.

We compare DiffKD with several representative anomaly detection methods. Considering the prevalence of knowledge distillation frameworks in anomaly detection, we choose STPM [14], DeSTSeg [16], and KR-FP [17] as competitive baselines, all of which adopt teacher–student architectures and learn compact feature representations for normal samples. We also include PatchCore [9], a typical memory-based method that stores prototypical normal patterns for efficient anomaly localization, providing a distinct and complementary paradigm to distillation-based pipelines. To further assess the effect of prior anomaly guidance on existing distillation baselines, DeSTSeg* and KR-FP* denote the corresponding models enhanced with four prior anomaly samples under λ=0.5. All methods are evaluated on the SUT-Crack dataset as well as the texture and object categories of MVTec AD. Qualitative results that intuitively illustrate the localization performance of different methods are visualized in Figure 8 and Figure 9. We repeat the experiments of our method five times with different random seeds to report the standard deviation.

On the SUT-Crack dataset, which contains thin cracks under cluttered backgrounds, STPM and PatchCore produce diffused heatmaps and confuse cracks with background noise. DeSTSeg and KR-FP reduce part of the background response but still miss discontinuous crack segments. With prior anomaly guidance, DeSTSeg* and KR-FP* provide more continuous responses than their original versions, yet residual noise and incomplete boundaries remain. In contrast, DiffKD preserves more complete crack contours while suppressing background interference, yielding the most precise localization in Figure 8.

Experimental results on MVTec AD further validate the competitive behavior of the proposed method across diverse anomaly types. As shown in Figure 9, STPM and PatchCore often generate diffused responses, while DeSTSeg and KR-FP improve spatial consistency but still show missed detections on subtle texture or structural defects. DeSTSeg* and KR-FP* benefit from prior anomaly samples and produce more compact activations than the original baselines. However, their responses can still deviate from ground-truth boundaries, especially for tiny object defects. DiffKD achieves cleaner anomaly activation, stronger background suppression, and better boundary alignment, indicating that the proposed discrepancy-guided distillation uses prior anomalies more effectively.

Quantitative results on SUT-Crack and MVTec AD are reported in Table 2, Table 3 and Table 4. For MVTec AD, mean I-AUC is first summarized, followed by category-wise P-AUC, AP, PRO, IAP, and IAP@90 to show performance variation across all 15 categories.

On SUT-Crack, PatchCore and STPM obtain competitive P-AUC values but weak AP and IAP, showing that their high pixel ranking does not translate into reliable crack localization. DeSTSeg and KR-FP improve instance detection, while DeSTSeg* and KR-FP* further raise AP, IAP, PRO, and I-AUC, confirming the value of limited prior anomaly guidance. DiffKD still outperforms both the original and prior-guided baselines on every metric, with clear gains over KR-F* in IAP@90 (18.9% vs. 15.8%) and PRO (86.0% vs. 82.1%). These results indicate stronger false-positive control and better preservation of thin crack structures.

Table 3 compares image-level anomaly classification on MVTec AD. All recent baselines achieve high I-AUC, showing that image-level discrimination is less difficult than accurate localization on this dataset. DiffKD still obtains the highest mean I-AUC (99.1%), slightly outperforming KR-FP and KR-FP*. This result indicates that the proposed anomaly-guided distillation does not sacrifice image-level recognition while improving pixel- and instance-level localization.

Table 4 reports per-category localization and instance-detection results on MVTec AD. Compared with DeSTSeg and KR-FP, DeSTSeg* and KR-FP* generally improve AP, PRO, IAP, and IAP@90, showing that four prior anomaly samples also benefit existing distillation baselines. DiffKD further achieves the best total averages of 99.1% P-AUC, 96.7% PRO, 81.9% IAP, and 66.5% IAP@90, and remains competitive in AP. The improvement is more evident on instance-level metrics than on P-AUC, because P-AUC is already near saturation whereas IAP and IAP@90 are more sensitive to false positives and missed small defects. For object categories, DiffKD gives the best object-average pixel-level and instance-level results, with clear advantages on bottle, metal nut, pill, transistor, and zipper. For texture categories, it also obtains the best average performance and strong results on carpet, leather, tile, and wood. DeSTSeg* and KR-FP* narrow the gap to DiffKD in several categories, especially where prior anomaly appearance is close to the test defects, but they remain less stable on tiny structures and repetitive textures. Overall, Table 4 shows that DiffKD uses prior anomalies more effectively than directly enhancing DeSTSeg or KR-FP, while the remaining category-specific weaknesses motivate the failure-case discussion in Section 5.

### 4.3. Justification of Training Strategy

**Figure 10:**
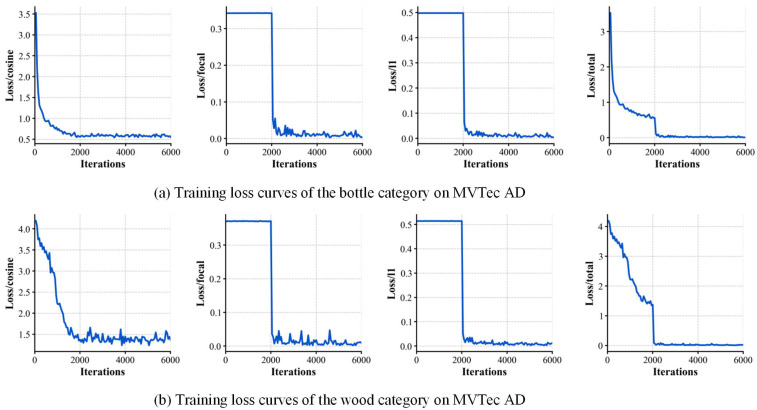
Training loss curves on MVTec AD.

To verify the rationality and effectiveness of the proposed training strategy, we visualize and analyze the dynamic evolution of loss functions during model training on two representative categories from the MVTec AD dataset. As illustrated in Figure 10, the training loss curves are provided for the bottle object category in Figure 10a and the wood texture category in Figure 10b, respectively, depicting the iterative variation of cosine loss, focal loss, L1 loss, and the overall total loss.

The model is trained under a well-designed two-stage optimization mechanism. In the early training phase (within the first 2000 iterations), optimization is exclusively dedicated to the abnormal discrepancy-guided denoising student network, which is updated solely via cosine loss, whereas the segmentation branch remains frozen and uninvolved in parameter updating. Consequently, cosine loss declines rapidly and dominantly governs the overall decreasing trend of total loss during this period. In contrast, focal loss and L1 loss, which are directly linked to the segmentation network, stay nearly constant since the segmentation module has not yet been activated for training. After the iteration count reaches 2000, training proceeds to the second stage, where the segmentation network is formally incorporated into joint optimization. At this point, both focal loss and L1 loss start to decrease steadily. With cosine loss continuing to converge and the segmentation-related losses declining synchronously, the total loss is further reduced consistently across both categories. Such a progressive loss pattern intuitively demonstrates the continuous enhancement of the model’s defect segmentation capability, which substantiates the efficacy of the proposed segmentation optimization scheme. Notably, the consistent convergence behavior observed on both the bottle rigid object and wood complex texture categories validates the strong stability and generalization of the phased training strategy. The core merit of this decoupled two-stage paradigm is that it effectively disentangles high-level normal feature learning from pixel-wise defect localization, thus eliminating task-level interference between feature representation learning and dense prediction. As a result, the model achieves stable convergence on diverse types of anomaly detection tasks while maintaining superior detection and segmentation performance, which further confirms the superiority and robustness of the proposed training mechanism.

### 4.4. Ablation Study

To comprehensively validate the effectiveness, rationality, and robustness of the proposed framework, a series of systematic ablation experiments were conducted on the MVTec AD dataset. First, we investigated the impact of varying numbers of prior anomaly samples on model performance and performed a sensitivity analysis of the lambda parameter in the CASG method to evaluate its robustness. Second, ablation studies were performed to verify the complementary contributions of the two core components (PA-ObjAug and CDGM) to the overall detection performance, clarifying their respective roles in enhancing anomaly localization and feature representation. Third, experiments were carried out to evaluate the adaptability of different input features to the segmentation task, ensuring the rationality of feature selection and its compatibility with the proposed method. Fourth, further ablation was conducted to explore the impact of different internal components within the CDGM module, identifying the key factors that drive its ability to capture differential anomaly features. Finally, the influence of PA-ObjAug dimensions on the performance of the CASG was investigated, optimizing the hyperparameter setting and verifying the generalization of PA-ObjAug in adjusting feature representation.

#### 4.4.1. Prior Anomaly Number Ablation of the CASG

Table 5 clearly presents the detection performance on MVTec AD when the number of prior anomaly samples per category varies among 1, 2, 4, and 8. The results show that increasing the number of prior anomalies from one to four steadily improves localization and instance-level detection, confirming that real anomaly guidance benefits CASG. The four-sample setting corresponds to the default configuration used in Table 4 and achieves the best overall balance across P-AUC, AP, IAP, IAP@90, and PRO. Although eight prior samples further improve AP slightly, the overall gain is limited relative to the additional annotation cost. Therefore, four prior anomalies are adopted as the default setting.

#### 4.4.2. λ Ratio Sensitivity Analysis of the CASG

We also conduct a sensitivity analysis on MVTec AD for the CASG mixing ratio, which controls the probability of selecting PA-ObjAug rather than SAG during online anomaly generation. Specifically, lambda is varied among 0.1, 0.3, 0.5, 0.7, and 0.9 to change the balance between real prior anomaly augmentation and synthetic anomaly generation. A smaller value emphasizes synthetic anomaly diversity, whereas a larger value emphasizes the morphology and appearance of limited real anomalies. The results are shown in Table 6.

As shown in Table 6, the best overall performance is obtained when λ=0.5, with 99.1% P-AUC, 80.7% AP, 81.9% IAP, 66.5% IAP@90, 96.7% PRO, and 99.1% I-AUC. When λ is small, CASG relies more on SAG, which increases synthetic diversity but weakens the use of real prior anomaly morphology; this leads to lower PRO and instance-level precision. When λ is large, PA-ObjAug dominates the generated samples, but the limited number of prior anomalies may reduce appearance diversity and cause lower AP and IAP@90. The balanced setting λ=0.5 combines realistic defect structures from PA-ObjAug with diverse synthetic variations from SAG, yielding the most stable pixel-level and instance-level performance. Therefore, λ=0.5 is adopted as the default mixing ratio.

#### 4.4.3. Effectiveness Verification of PA-ObjAug and CDGM

**Figure 11:**
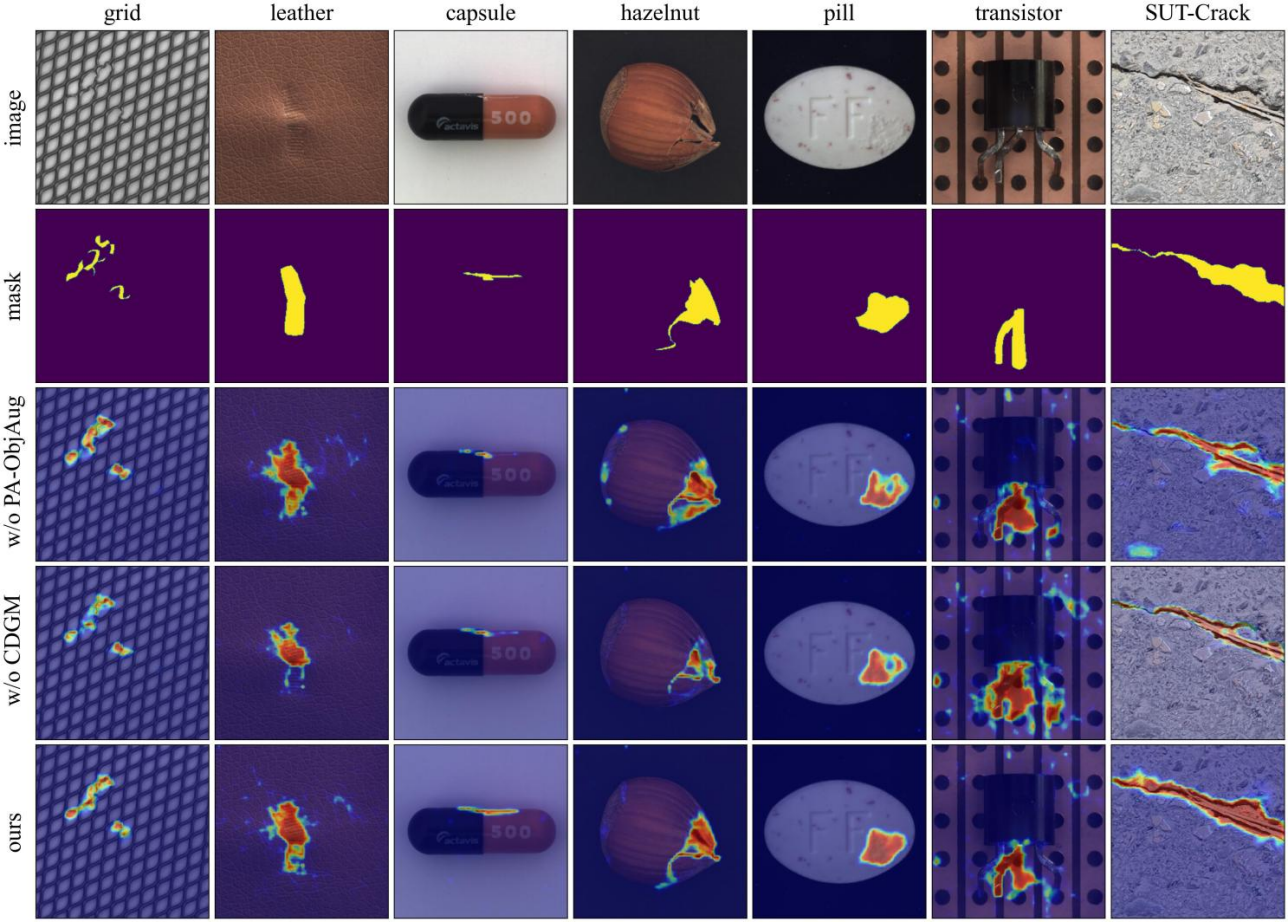
Qualitative visualization comparison of ablation studies on MVTec AD.

We evaluate PA-ObjAug and CDGM on MVTec AD using three variants: w/o PA-ObjAug, w/o CDGM, and the full model. Table 7 shows that removing either module reduces performance. Removing CDGM causes a larger drop, indicating that discrepancy guidance is central to the framework. Figure 11 further shows that PA-ObjAug reduces missed detections, while CDGM improves localization consistency.

#### 4.4.4. Adaptability of Different Input Features to the Segmentation Task

This section evaluates three feature combinations for the segmentation network:

1. concatenated-ST input: Features extracted from the teacher network and the student network are concatenated along the channel dimension and directly fed into the segmentation network;
2. cosine-distance input: Based on multi-scale feature maps derived from the teacher and student networks, the cosine similarity calculated across different scales is resampled to a unified scale, which serves as the input to the segmentation network to enhance the utilization of prior knowledge;
3. concatenated-STDiff input: Features from the teacher network, the student network, and the discrepancy-guided feature are concatenated holistically along the channel dimension, thereby maximizing the retention of information extracted by both the teacher and student networks, including both normal feature representations and abnormal discrepancy cues.

Table 8 shows that input feature selection affects segmentation performance. The proposed input achieves the best AP, IAP, IAP@90, PRO, and I-AUC, indicating that combining teacher–student discrepancies with reconstructed features provides more useful localization cues than using teacher–student concatenation or cosine distance alone.

#### 4.4.5. The Impact of Different Components in the CDGM Module

We analyze CDGM from two aspects: the contribution of its internal components and the ratio between amplitude and cosine discrepancies. Table 9 reports the component ablation, and Table 10 reports the ratio sensitivity.

Table 9 shows that $\Delta amp^{i}$, $\Delta \cos^{i}$, and CA are complementary. Using all three components gives the best overall result. Removing CA mainly weakens PRO and I-AUC, while using only one discrepancy term reduces instance-level performance, especially under the IAP@90 criterion.

As shown in Table 10, the 0.5:0.5 ratio achieves the best overall performance, reaching 99.1% P-AUC, 80.7% AP, 81.9% IAP, 66.5% IAP@90, 96.7% PRO, and 99.1% I-AUC. When the ratio is biased toward cosine discrepancy (0.2:0.8 or 0.4:0.6), P-AUC and I-AUC remain high, but IAP@90 decreases, indicating weaker instance localization under strict recall constraints. When the ratio is biased toward amplitude discrepancy (0.6:0.4 or 0.8:0.2), AP and IAP also decline, suggesting that magnitude-only differences are insufficient to distinguish subtle anomalous patterns from normal feature variations. Therefore, the balanced 0.5:0.5 setting is adopted because amplitude discrepancy captures response intensity changes, whereas cosine discrepancy captures feature-orientation changes; combining them equally provides a more stable anomaly representation.

#### 4.4.6. Impact of PA-ObjAug Dimensions on CASG Performance

Table 11 evaluates the contribution of angle, scale, and shape augmentation in PA-ObjAug together with SAG. Adding each dimension improves performance over SAG alone, and the full configuration achieves the best result. This indicates that the three augmentation dimensions are complementary.

### 4.5. Visualization of Intermediate Feature Maps

**Figure 12:**
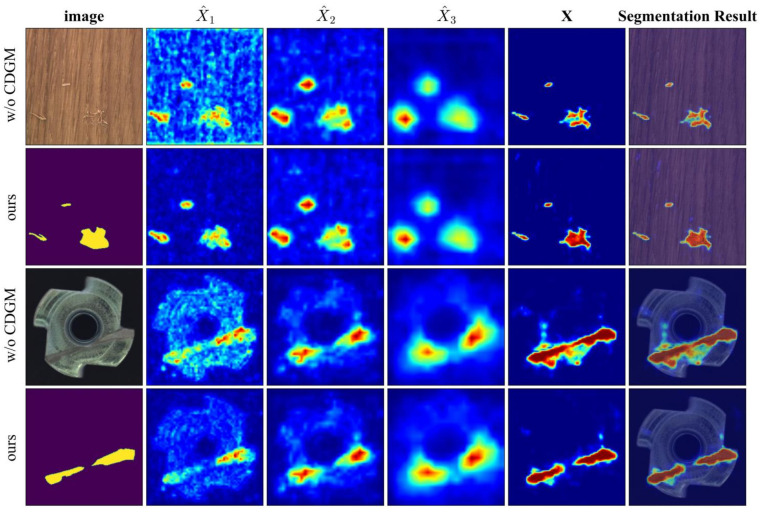
Comparison of deep features for anomaly detection. Warmer colors indicate stronger anomaly responses, whereas cooler colors indicate weaker responses.

Figure 12 compares intermediate discrepancy features ${\hat{X}}_{1}$, ${\hat{X}}_{2}$, and ${\hat{X}}_{3}$ with and without CDGM. Both variants can highlight anomaly-related regions, but the model without CDGM retains stronger background responses and edge noise. With CDGM, non-anomalous responses are more suppressed, producing cleaner multi-scale features for segmentation.

## 5. Discussion

### 5.1. Analysis of Failure Cases

**Figure 13:**
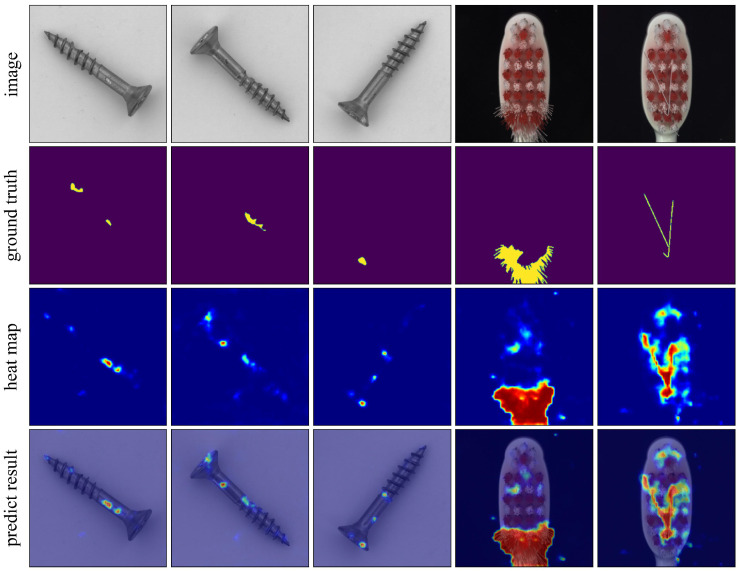
Failure cases analysis of the DiffKD method on MVTec AD. Warmer colors indicate stronger anomaly responses, whereas cooler colors indicate weaker responses.

Figure 13 presents representative failure cases on screws and toothbrushes.

For screw samples, subtle thread deformations may produce weak and discontinuous activations, while normal threads can trigger false responses. For toothbrush samples, dense bristle textures may cause over-segmentation. These cases indicate that periodic or densely repeated structures remain challenging. Future improvements may introduce boundary-aware constraints for periodic components, texture-consistency regularization for repeated patterns, or multi-scale attention in the segmentation branch.

### 5.2. Operating Efficiency

To evaluate deployability, we report inference latency and model size on the capsule category of MVTec AD. All methods are tested on the same platform: an Intel(R) Core(TM) i9-14900HX CPU, an NVIDIA GeForce RTX 4080 laptop GPU with 16 GB VRAM, and 32 GB RAM.

Table 12 summarizes inference time and model size. DiffKD requires 90 ms per image, close to DeSTSeg and KR-FP and faster than PatchCore. Its model size is 136 MB, comparable to DeSTSeg and KR-FP. These results indicate moderate computational cost for the reported accuracy.

### 5.3. Future Directions

The current study leaves two directions for future work. First, stronger distillation objectives, such as attention-based or adversarial distillation, may further improve student reconstruction. Second, a single ResNet-18 teacher may not capture both global semantics and fine local details. Multi-teacher distillation or transformer-based teachers such as DINOv2 may improve fine-grained anomaly detection.

## 6. Conclusions

This paper presents DiffKD, an anomaly discrepancy-guided knowledge distillation framework for image anomaly detection. The method uses normal teacher features as reconstruction targets and anomaly-derived discrepancies as guidance signals through CDGM. CASG further combines prior anomaly augmentation with synthetic anomaly generation to enrich training samples. Experiments on SUT-Crack and MVTec AD show that DiffKD improves localization while maintaining moderate computational cost. On MVTec AD, DiffKD achieves 80.7% AP and 81.9% IAP, showing competitive performance among the representative methods evaluated in this study. Remaining limitations on periodic and dense textures suggest future work on structural priors and stronger teacher models.

## Figures and Tables

**Table 1:** Layer correspondence between the teacher network and the denoising student network.

Feature Level	Teacher Output	Student Decoder Output	CDGM Placement
1/4 resolution	Block1, 64 channels	$S_{D}^{1}$, 64 channels	Applied before shallow reconstruction
1/8 resolution	Block2, 128 channels	$S_{D}^{2}$, 128 channels	Applied before middle reconstruction
1/16 resolution	Block3, 256 channels	$S_{D}^{3}$, 256 channels	Applied before deep reconstruction
1/32 resolution	Block4 removed	Bottleneck feature $S_{E}^{4}$	Not used as teacher target

**Table 2:** Quantitative analysis of the anomaly detection results (%) on SUT-Crack.

Method	P-AUC	AP	IAP	IAP@90	PRO	I-AUC
PatchCore [9]	91.5	28.7	8.7	4.6	72.1	80.3
STPM [14]	92.5	26.5	2.6	5.2	70.5	78.7
DeSTSeg [16]	86.8	37.1	34.7	4.6	68.5	92.7
KR-FP [17]	89.1	40.6	38.5	5.0	73.5	94.5
DeSTSeg*	91.6	59.8	60.2	12.1	79.2	95.6
KR-FP*	93.2	62.3	61.9	15.8	82.1	97.2
Ours	93.6±0.4	63.4±1.2	63.7±2.1	18.9±3.4	86.0±2.4	98.7±1.1

**Table 3:** Quantitative comparison of I-AUC (%) on MVTec AD.

STPM	PatchCore	DeSTSeg	KR-FP	DeSTSeg*	KR-FP*	Ours
95.1	98.5	98.4	98.9	98.6	98.9	99.1±0.2

**Table 4:** Per-category quantitative results (%) on MVTec AD. (**A**) reports pixel-level P-AUC/AP/PRO, and (**B**) reports instance-level IAP/IAP@90. For Ours, category-wise results are reported as mean ± std. The best performance for each category is shown in bold.

(A) Pixel-level (P-AUC/AP/PRO)							
**Category**	**STPM**	**PatchCore**	**DeSTSeg**	**KR-FP**	DeSTSeg*	KR-FP*	**Ours**
Bottle	98.8/80.6/96.4	98.9/80.1/96.0	99.2/90.3/96.6	99.2/91.0/96.8	99.0/92.2/**98.0**	**99.4**/93.4/97.4	**99.4_±0.2_/94.0_±1.3_**/97.6_±1.2_
Cable	94.8/58.0/84.3	**98.8**/70.0/**94.7**	97.3/60.4/86.4	96.9/65.7/89.7	97.9/**73.0**/91.4	97.9/71.4/92.7	95.8_±0.3_/71.5_±1.2_/89.1_±2.1_
Capsule	98.2/35.9/93.9	99.1/48.1/95.9	**99.2**/59.1/94.2	**99.2**/59.1/96.4	98.8/55.2/94.7	98.9/**59.7**/96.3	**99.2_±0.1_**/54.7_±1.6_/**97.4_±1.8_**
Hazelnut	98.9/60.3/96.7	99.0/61.5/96.2	99.6/88.4/97.6	99.6/87.8/97.7	98.6/81.5/97.9	**99.7/91.6/98.1**	99.4_±0.4_/84.1_±1.5_/**98.1_±1.1_**
Metal nut	97.2/79.3/94.2	98.8/88.8/95.7	98.6/93.5/95.0	99.1/93.8/95.8	**99.7**/98.1/97.2	**99.7/98.4**/96.8	**99.7_±0.2_**/98.0_±0.7_/**97.5_±1.6_**
Pill	94.7/63.3/93.3	98.2/78.7/96.2	98.7/83.1/95.3	99.0/85.7/96.3	99.5/94.1/96.3	99.5/94.4/96.0	**99.7_±0.2_/95.0_±1.8_/98.2_±0.8_**
Screw	98.6/26.9/93.9	**99.5**/41.4/**97.3**	98.5/58.7/92.5	99.2/56.9/95.1	99.2/55.1/96.3	98.9/**59.9**/94.0	99.0_±0.5_/53.1_±3.1_/95.3_±3.0_
Toothbrush	98.9/48.8/92.0	98.9/51.6/91.0	99.3/75.2/94.0	**99.6/79.1**/95.1	99.5/78.4/93.7	99.4/77.4/91.9	99.4_±0.0_/75.1_±2.4_/**95.7_±1.6_**
Transistor	81.9/44.4/68.1	96.2/63.2/91.0	89.1/64.8/85.7	94.4/71.3/90.1	98.3/**88.7**/95.3	98.5/85.9/93.3	**98.8_±0.3_**/88.0_±1.3_/**95.9_±2.1_**
Zipper	98.0/54.9/94.5	99.0/64.0/96.6	97.7/81.9/97.4	97.9/83.0/97.3	98.8/82.7/96.8	**99.6/88.3/98.0**	99.2_±0.2_/86.1_±1.1_/97.6_±0.9_
**Object Avg.**	96.0/55.2/90.7	98.6/64.8/95.1	97.7/75.5/93.5	98.3/76.2/95.0	98.9/79.9/95.8	**99.2/82.0**/95.4	99.0_±0.3_/80.0_±1.6_/**96.2_±1.6_**
Carpet	99.1/65.3/97.1	99.1/66.7/95.5	96.1/72.8/93.6	98.5/80.4/97.2	98.2/82.8/96.5	**99.3/87.3/97.9**	99.0_±0.2_/86.1_±1.6_/97.7_±1.4_
Grid	**99.1**/45.4/**97.0**	98.9/41.0/94.1	**99.1**/61.5/96.4	**99.1/62.0**/96.2	97.6/52.7/92.3	98.6/61.7/94.5	**99.1_±0.6_**/59.6_±2.4_/96.3_±2.3_
Leather	99.2/42.9/97.6	99.4/51.0/97.9	99.7/75.6/99.0	99.7/75.6/99.1	**99.8**/76.7/99.3	**99.8**/79.3/**99.5**	**99.8_±0.1_**/**81.1_±1.1_**/98.4_±0.3_
Tile	96.6/61.7/87.3	96.6/59.3/87.7	98.0/90.0/95.5	98.6/93.3/97.1	98.6/92.2/97.0	98.9/94.5/**97.7**	**99.5_±0.5_/96.3_±1.4_**/97.5_±1.1_
Wood	95.2/47.0/91.9	95.1/52.3/91.3	99.1/85.2/96.1	**99.2**/85.2/96.5	99.1/86.2/97.0	98.5/87.4/**97.8**	98.9_±0.3_/**87.8_±1.0_**/97.6_±0.6_
**Texture Avg.**	97.8/52.5/94.2	97.8/54.1/93.3	98.4/77.0/96.1	98.2/76.9/97.2	98.7/78.1/96.4	99.0/82.0/**97.5**	**99.3_±0.3_/82.2_±1.5_/97.5_±1.1_**
**Total Avg.**	96.6/54.3/91.9	98.4/61.2/94.5	97.9/76.0/94.4	98.6/78.0/95.8	98.8/79.3/96.0	99.1/**82.0**/96.1	**99.1_±0.3_**/80.7_±1.6_/**96.7_±1.5_**
**(B) Instance-level (IAP/IAP@90)**							
**Category**	**STPM**	**PatchCore**	**DeSTSeg**	**KR-FP**	DeSTSeg*	KR-FP*	**Ours**
Bottle	83.2/73.3	81.8/70.1	91.7/87.4	92.9/86.9	**95.3**/87.4	93.9/89.2	94.6_±1.2_/**91.0_±2.3_**
Cable	54.9/17.2	69.2/**50.6**	51.3/30.0	60.2/34.9	69.7/44.1	69.5/46.4	**71.5_±1.8_**/26.5_±3.0_
Capsule	37.2/17.9	44.2/26.9	44.6/23.5	**57.5/40.3**	49.6/27.7	56.7/30.6	56.7_±1.5_/38.1_±2.6_
Hazelnut	64.8/56.2	63.8/52.5	88.1/79.6	86.9/76.5	88.8/77.3	**90.4/83.0**	89.1_±2.4_/80.9_±5.1_
Metal nut	83.4/81.7	90.1/84.6	92.3/83.1	95.0/88.3	96.8/91.8	96.0/92.2	**97.1_±1.3_/93.1_±2.8_**
Pill	72.0/45.5	82.7/63.5	77.8/38.9	88.1/74.9	87.8/59.7	87.2/62.7	**94.3_±2.1_/84.5_±4.6_**
Screw	24.4/4.2	38.4/16.3	53.8/6.4	52.7/5.3	50.8/9.2	**56.0/13.2**	48.4_±4.2_/11.0_±3.5_
Toothbrush	41.9/23.4	40.4/22.1	60.6/42.9	62.8/**45.9**	63.0/27.7	59.6/20.5	**64.1_±1.8_**/40.9_±2.9_
Transistor	53.4/8.5	69.9/36.8	83.8/69.9	82.6/65.1	92.1/**81.0**	85.8/66.3	**93.4_±1.6_**/78.8_±2.1_
Zipper	59.1/46.6	66.0/52.4	88.5/74.5	89.0/76.7	88.2/71.4	90.9/79.9	**91.6_±2.0_/83.5_±2.8_**
**Object Avg.**	57.5/37.4	64.6/47.6	73.2/53.6	76.8/59.5	78.2/57.7	78.6/58.4	**80.1_±2.0_/62.8_±3.2_**
Carpet	68.4/52.2	64.4/43.7	76.0/30.8	87.6/70.4	91.4/82.5	91.3/82.5	**92.1_±1.3_/82.8_±1.5_**
Grid	45.7/21.0	39.1/15.6	**62.2/45.1**	57.8/30.2	54.7/14.9	61.8/35.6	60.2_±1.6_/35.4_±2.3_
Leather	46.2/24.9	50.1/30.1	78.1/67.3	81.2/70.4	80.0/68.9	83.5/71.5	**87.2_±1.2_/75.7_±1.6_**
Tile	62.9/55.3	60.0/52.1	97.4/**93.9**	96.2/92.6	96.0/90.2	**98.0**/93.7	96.6_±1.8_/91.0_±2.3_
Wood	56.0/35.4	59.7/35.6	73.3/57.0	89.5/82.8	91.2/86.5	**94.5/88.1**	91.3_±1.4_/84.3_±3.4_
**Texture Avg.**	55.8/37.8	54.7/35.4	77.4/58.8	82.5/69.3	82.7/68.6	**85.8/74.3**	85.5_±1.5_/73.8_±2.2_
**Total Avg.**	56.9/37.5	61.3/43.5	74.6/55.4	78.7/62.7	79.7/61.4	81.0/63.7	**81.9_±1.8_/66.5_±2.9_**

**Table 5:** Ablation study on the number of prior anomaly samples on MVTec AD (%).

Prior Anomaly Number	P-AUC	AP	IAP	IAP@90	PRO	I-AUC
1	97.7	76.8	79.4	61.3	94.7	98.6
2	98.3	78.8	80.0	62.5	95.3	98.7
4	99.1	80.7	81.9	66.5	96.7	99.1
8	98.7	81.0	80.7	63.3	95.3	98.6

**Table 6:** λ ratio sensitivity analysis on MVTec AD (%).

λ	P-AUC	AP	IAP	IAP@90	PRO	I-AUC
0.1	98.3	80.0	80.7	64.6	94.3	91.7
0.3	98.5	79.2	79.1	61.8	95.1	98.9
0.5	99.1	80.7	81.9	66.5	96.7	99.1
0.7	97.3	77.5	78.5	62.2	93.8	97.3
0.9	98.8	80.6	81.5	63.9	96.3	99.0

**Table 7:** Ablation analysis of our method on MVTec AD (%).

Method	P-AUC	AP	IAP	IAP@90	PRO	I-AUC
w/o SAG	97.8	75.2	76.4	58.6	94.1	98.2
w/o PA-ObjAug	98.4	76.2	79.2	62.1	95.3	98.4
w/o CDGM	97.8	75.2	76.4	58.6	94.1	98.2
Ours	99.1	80.7	81.9	66.5	96.7	99.1

**Table 8:** Impact (%) of different segmentation network inputs on experimental results.

Method	P-AUC	AP	IAP	IAP@90	PRO	I-AUC
concatenated-ST	98.2	77.8	77.7	57.3	93.5	97.3
cosine-distance	98.8	76.4	78.0	62.2	95.5	98.8
concatenated-STDiff	98.3	78.4	77.6	56.8	94.2	97.4
Ours	99.1	80.7	81.9	66.5	96.7	99.1

**Table 9:** The impact (%) of the different components in the CDGM module. A checkmark indicates that the corresponding component is used.

$\Delta {\mathrm{amp}}^{i}$	$\Delta \cos^{i}$	CA	P-AUC	AP	IAP	IAP@90	PRO	I-AUC
✓		✓	98.8	79.8	80.3	61.5	95.8	98.6
	✓	✓	98.8	76.4	78.0	62.2	95.5	98.8
✓	✓		98.5	79.3	80.5	65.2	95.6	98.0
✓	✓	✓	99.1	80.7	81.9	66.5	96.7	99.1

**Table 10:** The impact (%) of the weight ratio between different differential features.

$\Delta {\mathrm{amp}}^{i}$	$\Delta \cos^{i}$	P-AUC	AP	IAP	IAP@90	PRO	I-AUC
0.2	0.8	98.8	79.0	80.1	63.4	96.1	99.0
0.4	0.6	98.6	79.3	79.9	60.9	95.5	99.0
0.5	0.5	99.1	80.7	81.9	66.5	96.7	99.1
0.6	0.4	98.9	80.0	80.2	62.6	96.0	98.9
0.8	0.2	98.9	80.2	80.5	63.1	96.1	98.9

**Table 11:** Ablation study on the contribution of each augmentation dimension in PA-ObjAug on MVTec AD (%). A checkmark indicates that the corresponding component is used.

Augmentation Dimensions				Performance Metrics					
**Angle**	**Scale**	**Shape**	**SAG**	**P-AUC**	**AP**	**IAP**	**IAP@90**	**PRO**	**I-AUC**
			✓	97.8	75.2	76.4	58.6	94.1	98.2
✓			✓	98.5	79.0	79.8	63.5	94.9	98.5
	✓		✓	98.0	77.0	78.0	59.2	95.0	98.2
		✓	✓	98.4	79.5	80.8	63.1	95.7	98.6
✓	✓		✓	98.5	80.1	80.0	62.3	95.4	98.4
✓		✓	✓	98.5	78.2	78.6	61.8	95.0	98.0
	✓	✓	✓	98.8	80.4	80.6	64.2	96.3	98.9
✓	✓	✓	✓	99.1	80.7	81.9	66.5	96.7	99.1

**Table 12:** Inference time and model size comparison of different anomaly detection methods.

Method	Inference Time (ms)	Model Size (Mb)
PatchCore [9]	98	65
STPM [14]	25	50
DeSTSeg [16]	90	134
KR-FP [17]	92	138
Ours	90	136

## Data Availability

The dataset used in this work consists of SUT-Crack and MVTec AD. The SUT-Crack dataset is publicly available at https://doi.org/10.1016/j.dib.2023.109642. The MVTec AD dataset generated during and/or analyzed during the current study is available at https://www.mvtec.com/company/research/datasets/mvtec-ad (accessed on 25 June 2026).

## References

[1] Kumari S., Prabha C., Karim A., Hassan M.M., Azam S. (2024). A Comprehensive Investigation of Anomaly Detection Methods in Deep Learning and Machine Learning: 2019–2023. IET Inf. Secur..

[2] Liu X., Wang J., Leng B., Zhang S. (2024). Dual-Modeling Decouple Distillation for Unsupervised Anomaly Detection. arXiv.

[3] Qi P., Chai L., Ye X. (2024). Unsupervised Industrial Anomaly Detection Based on Feature Mask Generation and Reverse Distillation. Chin. J. Inf. Fusion.

[4] Liu X., Wang J., Leng B., Zhang S. (2025). Unlocking the Potential of Reverse Distillation for Anomaly Detection. Proc. AAAI Conf. Artif. Intell..

[5] Sabouri M., Sepidbar A. (2023). SUT-crack: A comprehensive dataset for pavement crack detection across all methods. Data Brief..

[6] Bergmann P., Fauser M., Sattlegger D., Steger C. (2019). MVTec AD: A comprehensive real-world dataset for unsupervised anomaly detection. 2019 IEEE/CVF Conference on Computer Vision and Pattern Recognition (CVPR).

[7] Rudolph M., Wehrbein T., Rosenhahn B., Wandt B. (2022). Fully convolutional cross-scale-flows for image-based defect detection. Proceedings of the IEEE/CVF Winter Conference on Applications of Computer Vision.

[8] Rudolph M., Wandt B., Rosenhahn B. (2021). Same same but DifferNet: Semi-supervised defect detection with normalizing flows. 2021 IEEE Winter Conference on Applications of Computer Vision (WACV).

[9] Roth K., Pemula L., Zepeda J., Schölkopf B., Brox T., Gehler P. (2022). Towards total recall in industrial anomaly detection. Proceedings of the IEEE/CVF Conference on Computer Vision and Pattern Recognition.

[10] Defard T., Setkov A., Loesch A., Audigier R. (2021). Padim: A patch distribution modeling framework for anomaly detection and localization. International Conference on Pattern Recognition.

[11] Bergmann P., Fauser M., Sattlegger D., Steger C. (2020). Uninformed students: Student-teacher anomaly detection with discriminative latent embeddings. Proceedings of the IEEE/CVF Conference on Computer Vision and Pattern Recognition.

[12] Deng H., Li X. (2022). Anomaly Detection via Reverse Distillation from One-Class Embedding. Proceedings of the IEEE/CVF Conference on Computer Vision and Pattern Recognition (CVPR).

[13] Salehi M., Sadjadi N., Baselizadeh S., Rohban M.H., Rabiee H.R. (2021). Multiresolution knowledge distillation for anomaly detection. 2021 IEEE/CVF Conference on Computer Vision and Pattern Recognition (CVPR).

[14] Wang G., Han S., Ding E., Huang D. (2021). Student-teacher feature pyramid matching for anomaly detection. arXiv.

[15] Yamada S., Hotta K. (2022). Reconstruction student with attention for student-teacher pyramid matching. arXiv.

[16] Zhang X., Li S., Li X., Huang P., Shan J., Chen T. (2023). DeSTSeg: Segmentation guided denoising student-teacher for anomaly detection. 2023 IEEE/CVF Conference on Computer Vision and Pattern Recognition (CVPR).

[17] Zang Y., Lu A., Li B., Hu W. (2024). Revisiting segmentation-guided denoising student-teacher in anomaly detection. Vis. Comput..

[18] Liu X., Wang J., Leng B., Zhang S. (2024). Dual-Modeling Decouple Distillation for Unsupervised Anomaly Detection. Proceedings of the 32nd ACM International Conference on Multimedia (MM ’24).

[19] Huang W., Xu Z., Wan R., Yang X., Zhang B. (2025). KD-KI: Knowledge distillation with knowledge infusion for anomaly detection and localization. Neurocomputing.

[20] Hu R., Yi J., Li X., Chen A., Han G. (2026). Efficient industrial anomaly detection via cross-scale distillation with enhanced feature compression. Pattern Recognit..

[21] Li L.H., Tanone R. (2023). Improving robustness using MixUp and CutMix augmentation for corn leaf diseases classification based on ConvMixer architecture. J. ICT Res. Appl..

[22] DeVries T., Taylor G.W. (2017). Improved regularization of convolutional neural networks with cutout. arXiv.

[23] Yun S., Han D., Chun S., Oh S.J., Yoo Y., Choe J. (2019). CutMix: Regularization strategy to train strong classifiers with localizable features. 2019 IEEE/CVF International Conference on Computer Vision (ICCV).

[24] Lyu X., Tian Z., Zhao X., Han J., Cai Z., Chen Y. (2025). Multi-modal semi-supervised semantic segmentation for indoor scenes via adaptive CutMix and contrastive learning. Multimed. Syst..

[25] Liu W., Wang C., Zhang Y. (2025). Industrial surface defect detection by multi-scale Inpainting-GAN. Vis. Comput..

[26] Odena A., Olah C., Shlens J. (2016). Conditional image synthesis with auxiliary classifier GANs. arXiv.

[27] Chen X., Duan Y., Houthooft R., Schulman J., Sutskever I., Abbeel P. (2016). InfoGAN: Interpretable representation learning by information maximizing generative adversarial nets. arXiv.

[28] Lopez R., Regier J., Jordan M.I., Yosef N. (2018). Information constraints on auto-encoding variational bayes. arXiv.

[29] Yan H., Yeh H.M., Sergin N. (2019). Image-based process monitoring via adversarial autoencoder with applications to rolling defect detection. 2019 IEEE 15th International Conference on Automation Science and Engineering (CASE).

[30] Hu T., Zhang J., Yi R., Du Y., Chen X., Liu L., Wang Y., Wang C. (2023). AnomalyDiffusion: Few-Shot Anomaly Image Generation with Diffusion Model. arXiv.

[31] Jin Y., Peng J., He Q., Hu T., Wu J., Chen H., Wang H., Zhu W., Chi M., Liu J. (2024). DualAnoDiff: Dual-Interrelated Diffusion Model for Few-Shot Anomaly Image Generation. arXiv.

[32] Gui S., Gao B.-B., Wang C., Wu Y. (2025). Few-Shot Anomaly-Driven Generation for Anomaly Classification and Segmentation. arXiv.

[33] He H., Zhang J., Chen H., Chen X., Li Z., Chen X., Wang Y., Wang C., Xie L. (2023). DiAD: A Diffusion-based Framework for Multi-class Anomaly Detection. arXiv.

[34] Rother C., Kolmogorov V., Blake A. (2004). GrabCut: Interactive foreground extraction using iterated graph cuts. ACM Trans. Graph..

[35] Jaskowiak P., Costa I., Campello R. (2022). The area under the ROC curve as a measure of clustering quality. Data Min. Knowl. Discov..

[36] He K., Lu Y., Sclaroff S. (2018). Local descriptors optimized for average precision. Proceedings of the IEEE/CVF Conference on Computer Vision and Pattern Recognition (CVPR).

